# The canary in the coal mine: Continence care for people with dementia in acute hospital wards as a crisis of dehumanization

**DOI:** 10.1111/bioe.12446

**Published:** 2018-04-20

**Authors:** Paula Boddington, Katie Featherstone

**Keywords:** continence care, dementia, dignity, elder care, hospital care, nursing, personhood

## Abstract

Continence is a key moment of care that can tell us about the wider care of people living with dementia within acute hospital wards. The spotlight is currently on the quality of hospital care of older people across the UK, yet concerns persist about their poor treatment, neglect, abuse, and discrimination within this setting. Thus, within hospitals, the care of people living with dementia is both a welfare issue and a human rights issue. The challenge of continence care for people living with dementia can be seen as the ‘canary in the coal mine’ for the unravelling of dignity within the acute setting. This paper draws on an ethnographic study within five hospitals in England and Wales, selected to represent a range of hospital types, geographies and socio‐economic catchments. Observational fieldwork was carried out over 154 days in acute hospitals known to admit large numbers of people living with dementia. This paper starts to fill the gap between theory and data by providing an in‐depth ethnographic analysis examining the ways in which treatment *as a person* is negotiated, achieved or threatened. We examine how the twin assaults on agency of a diagnosis of dementia and of incontinence threaten personhood. The acute threats to this patient group may then act to magnify perils to treatment as a person. Our findings suggest that personal dignity and the social construction of moral personhood are both threatened and maintained in such a setting. We show how empirical ethnographic data can lend weight to, and add detail to, theoretical accounts of moral personhood and dignity.

## 1 BACKGROUND TO THE ETHICAL ISSUES: THE CARE OF PEOPLE WITH DEMENTIA IN ACUTE HOSPITAL WARDS

The spotlight is currently on the quality of hospital care of older people across the UK. Concerns persist about poor treatment, neglect, abuse, and discrimination of older people in acute hospitals.^1^ Recent reports identify widespread poor dementia care, with broad variation in quality, meaning that people living with dementia are ‘likely to experience poor care at some point along their care pathway’.^2^

A diagnosis of dementia is associated with increased risk of acute hospitalization,^3^ with UTIs,^4^ pneumonia,^5^ nutritional disorders,^6^ and hip fractures^7^ being the principal causes of admission. People living with dementia occupy 25% of acute hospital beds and account for around 3.2 million bed days per year. However, some acute hospitals report 40 to 50% of their patients have dementia.^8^

### 1.1 Hospital care, dementia and incontinence

The care of people living with dementia within hospitals is not only a welfare issue but also a human rights issue. An important area of everyday care is continence care, which has been largely overlooked in research, and which we will argue is often rendered invisible. ‘Toilet Access and Use’ is a key marker of human rights and dignity.^9^ A lack of dignity in the personal care needs of older patients is a recurrent theme within a number of reviews and enquiries.^10^

A Public Inquiry addressed the underlying systemic issues and identified that ‘elderly and vulnerable patients … were deprived of dignity and respect. Some patients had to relieve themselves in their beds when they (were) offered no help to get to the bathroom. Some were left in excrement stained sheets and beds’.^11^ Witnesses to a House of Lords House of Commons Joint Committee reported similar findings including people not being allowed to use the toilet in private.^12^ Various reports find delays in personal care assistance, insufficient patient privacy when receiving care and using the toilet, and a dissatisfaction with toilet facilities.^13^

### 1.2 The social and organizational context of care

A body of qualitative social science research highlights the importance of the social and organizational context of care at ward level,^14^ the importance of relational work in delivering care quality within acute hospital wards,^15^ and the significance of the nursing role in identifying and promoting dignity for older people living with dementia.^16^

Given the increasing delegation of ‘hands‐on’ care in acute hospital wards to Healthcare Assistants (HCAs), it is important to focus on this less privileged and marginalized group,^17^ who can influence how care is organized, supervised and delivered.^18^ ‘Dirty work’, ‘elimination work’, ‘body work’ or ‘body labour’, paid work carried out on the bodies of others,^19^ and concerning the body's wastes is habitually regarded as low status, bordering on the polluted,^20^ and is often gendered.^21^ This work poses a serious threat to formal caregivers’ sense of self and status, with higher status workers distancing themselves from bodywork.^22^ Despite its obvious necessity, it is also work that is in many ways invisible,^23^ or at least treated as if it should be invisible, with body workers engaging in practices that hide ‘dirty work’ from others, for example drawing screens around the bed,^24^ or within patient bedrooms,^25^ protecting the dignity of both the patient and the workers. However, helping patients to use the toilet and to support the maintenance of dignity, wellbeing and quality of life is a core nursing role.^26^

## 2 INCONTINENCE AS A HALLMARK OF A DEHUMANIZED STATUS: AGENCY, PERSONHOOD, AND DIGNITY

Both dementia and incontinence are points of moral challenge. They both have the potential to elicit strong moral responses of care and concern, and conversely, strong aversion and stigma, which can occasion rejection and diminished moral and social status. Both theoretical work in moral philosophy and empirical work in the social sciences help to shed light on this ethical duality. To receive responses from others appropriate to one's standing as a moral person, one must be perceived in an appropriate way, including having an acceptable presence within the social world.

Philosophical accounts of personhood tend to focus upon mental capacities, but with an assumption of embodiment essential in the realization of the agency that is key to personhood.^27^ Agency is frequently understood in terms involving intentional action via successful control over one's body.^28^ A loss of mental and physical agency threatens the status of personhood.^29^

The accounts of personhood that we take to be most plausible note the social and relational aspects of personhood and the reciprocity of social interaction and acceptability.^30^ Hence, it is vital to consider how our ethical identity, autonomy and worth are constructed through how others see and respond to us. Philosophical work which looks closely at humanity as embodied beings and at the phenomenology of moral thought, has examined how our appearances to others and the precise and individual instantiation of embodiment are involved in being recognized and treated as a moral being.^31^ Likewise, there has been much recent discussion of the related concept of dignity with respect to older people and people living with dementia.^32^ Much of this notes distinctions between an absolute notion of dignity (what Nordenfelt describes as *Menschenwϋrde*) and relative or subjective notions where dignity is dependent on the awareness of others, such as dignity of merit, dignity of moral stature and dignity of identity. The behavior of others can therefore threaten these relative understandings of dignity.^33^

Dementia produces diminished mental capacity in respect of key aspects of personhood and agency: memory, planning, awareness of self and others. However, as well as challenges attributable to the condition itself, the wider cultural fear of dementia makes it additionally problematic for others to interact with the individual in ways that recognize their personhood.^34^ Control over the body both enables the realization of intentional action, and maintains the boundaries between the socially acceptable body and its socially unacceptable waste products through control over elimination. Note, such control is achieved through direct self‐knowledge of the body's promptings; and this is control which in normal circumstances requires managed access to private toilet facilities as the individual navigates their entry and exit from shared social view.

Such spoilers of personhood also threaten the acceptable inclusion into the social sphere and the reciprocity with others which acts to sustain one's presence in the moral community. Where incontinence and dementia both directly challenge physical and mental agency, and an acceptable social presence, there is a double negative feedback loop between diminished agency and the social construction of perceived personhood.

Work in social psychology about the conditions under which we recognize the humanity and moral claims of others demonstrates the ease with which dehumanization happens, and the influence that social hierarchy and role may have.^35^ Strains and stresses on the actor – such as time pressures, social pressures such as desire for obedience to authority, peer pressure, and other institutional issues – are significant factors, and these are likely to present themselves in the setting of acute hospital wards. Poor, uniform, or absent clothing, the absence of markers of individuality, and, tellingly, denial of adequate toilet facilities are among the markers of ready dehumanization.^36^ These findings reinforce the rationale for our inquiry. Here we are examining acute hospital wards; however, there is a significant body of work examining the ways in which long‐term community settings dignity can be influenced both by individuals within it and by external factors.^37^ Of particular relevance, one study based on interviews with people living within a long‐term community setting, reported that a key issue affecting their dignity was ‘the unrecognizable body’ and no longer being able to control their body and its functions, particularly their bladder and bowels.^38^

### 2.1 Articulating the enactment of personhood and threats to dignity

Work within the nursing and policy sphere concerning the care of people living with dementia draws heavily and centrally upon notions of personhood;^39^ but the notion of the person is often not articulated.^40^ A growing body of theoretical work examines personhood in dementia.^41^ Yet, empirically based work may lack analytic theory.^42^

Whilst there is little research examining continence care in the acute hospital setting, there is a larger body of research examining continence care and dignity for older people within long‐term community settings.^43^ Many of these studies cite the need for further empirical research especially in view of the difficulty of providing universally agreed definitions of dignity.^44^ Although there are few mentions of continence care within this literature, these references are generally critical of such care. For example, instances are described of residents being shouted at ‘you have to poop in the nappy’ during the night, rather than being assisted to get out of bed and to go to the bathroom.^45^

This work starts to fill this gap between theory and data^46^ by considering in‐depth ethnographic analysis examining the ways in which the recognition of personhood is negotiated, achieved, and threatened. We examine how the twin assaults on agency of dementia and incontinence threaten personhood. The acute threats to people living with dementia may then act to magnify perils to treatment as a person more generally. The hospital setting, with its distinct dominance hierarchies, can cast a focused light on some of the systemic, social and hierarchical elements of the construction and destruction of personhood and moral dignity.

## 3 METHODS

Our approach to ethnography is informed by the symbolic interactionist research tradition, which aims to provide an interpretive understanding of the social world.^47^ The value of this approach is the depth of understanding and theory generation it can provide.^48^ Our ethnographic approach enables us to understand how ward staff respond to the care needs of people living with dementia. Importantly, we also examine how staff account for and make sense of their responses to the care needs of people living with dementia in these contexts. Ethnography allows examination of the interplay between these factors.^49^

Purposive and maximum variation sampling was used to include five hospitals that represent hospital types (two large University teaching hospitals, two medium‐sized general hospitals and one smaller general hospital), and varying geographical and demographic locations throughout England and Wales. These hospitals represent a range of expertise and interventions in caring for people living with dementia.^50^

Across these sites, 154 days of observational fieldwork were carried out in acute hospital wards known to have a large number of people with dementia. Approximately 600,000 words of observational fieldnotes have been transcribed, cleaned and anonymized. Data collection focussed on the work of nurses and HCAs (Healthcare Assistants). These field notes were complemented by 436 ethnographic (during observation) interviews conducted in the course of the everyday life of the ward, taken from 155 participants including ward staff, carers and patients themselves. Detailed case studies were then conducted with 10 of these patients, observing care and speaking to the person and their family carers throughout their admission.

Data collection (observations and interviews) and analysis has been informed by the analytic tradition of grounded theory.^51^

Here, we focus on exploring the theme of continence care that emerged during fieldwork and analysis. We are only able to present a snapshot of our findings and focus on those illustrative of the barriers and enablers to dignity and recognition of agency and personhood.

Ethics Committee approval for the study was granted by the NHS Research Ethics Service (15/WA/0191). Substantial amendments to the study protocol were approved at a meeting of the Wales REC 3 committee on December 4, 2015. Substantial amendments to the study protocol were approved at a meeting of the Wales REC 3 committee on December 10, 2015. The study was accepted by NHS Research Permissions Wales on July 16, 2015, with NIHR CSP and West Midlands CRN on March 11, 2016 and with the Health Research Authority on May 27, 2016. Recruitment for the study was managed and recorded through the Central Portfolio Management System and closed on January 31, 2017. The committee has approved this research project for the purposes of the Mental Capacity Act 2005 and confirms that it meets the requirements of section 31 of the Act in relation to research carried out as part of this project on, or in relation to, a person who lacks capacity to consent to taking part in the project. All sites, individuals, and data collected have been anonymized and sorted in line with the Data Protection Act 1998, and NHS England Data Protection Policy 2014. Storage of the data is managed by the Cardiff University Information Security Framework Program.

## 4 ANALYSIS

### 4.1 Continence care is a significant part of everyday care in wards

Although continence care is a significant part of the everyday work of the ward, it is often covert, out of sight or concealed work. Continence care (staff) and continence needs (patients) are hard to speak about. However, the essential nature of continence care means that it does need to be spoken about, or a patient's needs must be recognized non‐verbally so that they can be acted on, including acknowledging problems such as mobility and helping patients to access toilets, to recognition of urgency or constipation. This starts the process of moving continence from a private locus of control to a more externalized locus of control. For people living with dementia who may have mobility or communication difficulties, this extends to needing to seek recognition of their needs and requiring permission.

Here this nurse recognizes that continence care is a difficult topic for all her patients to talk about, whether they have a dementia diagnosis or not. In response, she tells me (KF) that she prompts patients by asking them and by looking at their behaviour – agitation may be a sign that they need continence care:

During the shift the nurse comes over to me (KF) and chats about the dementia patients in her bay: ‘Because I have been off, I have spoken to the lady with dementia and the lady (a 75 year old woman admitted with a fractured hip and wrist), I had to ask her, I know she is on [medication] so it's likely she is constipated and I asked her, she can't say she is constipated, you have to ask. It turns out she hasn't been since she was admitted 6 days ago! I will get her a prescription. Similar with the dementia patients, they can't say they are constipated and although we have charts, you sort of know from their behaviour they are agitated and you can usually trace it back – if you give them an enema or suppositories or start them on laxatives’… . ‘This lady has no problems but she is constipated – she needs this for the pain and so I will give her a prescription – she's been in here 6 days and she hasn't mentioned it!’ [Site A, day 1]

However, she also has an expectation that people living with dementia should be able to recognize and communicate their continence care needs directly to staff.

#### 4.1.1 A cascading sense of indignity

When assistance is required that must take place at the bedside, there is a sense of embarrassment for the patient and also the ward team. This patient attempts to make a request for continence care that is discreet, finds it hard to discuss her continence problems (constipation) and finds it taxing to recover from this intimate encounter:

The patient (an 85 year old woman with a diagnosis of dementia who has spinal problems following a fall) is sitting in the chair with a glass of water on the trolley in front of her. When the HCA passes, she calls her over and says very quietly ‘Can I have a commode please’. The HCA gets it straight away and returns with the commode and closes the curtain around the bedside. It feels very calm and peaceful in this large bright room, the only sound the hum of the air con unit and the curtains have been closed for a long time. The patient is still using the commode and she has a long discussion with the team (the HCA and nurse who has joined them) about her constipation. Eventually the team pull the curtain back and she is tucked up in bed covered in a sheet. She is reading the large laminated menu and has her head down and hidden from view. She may be hiding behind it, I (KF) sense embarrassment. The team walk away and seem exhausted by that encounter. [Site D day 3]

This encounter is ‘behind the screen’, however, this sense of embarrassment following this intimate encounter which involved a group discussion with other members of staff, lingers for both the patient and the ward team.

#### 4.1.2 The private becomes open to control by others

The lines of privacy become altered in the context of the ward; people no longer have control over how others see them and it is no longer unusual for people to be viewed during intimate moments, or for their continence care needs to be delayed or interrupted by the competing priorities of others. Here the patient is sitting (dressed in a hospital gown) on a commode being wheeled to the bathroom when the medical team arrive and interrupt this to disclose complex and significant new information about her diagnosis and the implications for her care. This also signals a loss of control; dependent on others to facilitate her continence yet they can delay her, and the priorities of the medical team take precedence regardless of her continence needs or the inappropriateness of the timing and setting:

A HCA asks the patient (88 year old woman living with dementia who was admitted with a fractured hip) if she is ready for lunch – ‘Lunch? I've only just had breakfast’! She looks startled by that suggestion and responds that she would like to use the toilet. The HCA gets the commode and the bay nurse joins them and they ask the patient if she wants to use the commode or go to the bathroom. She decides on going to the bathroom and the HCA and the nurse helps her up out of the chair and as they do this they discuss what to do and decide to wheel her to the bathroom sitting (dressed in a hospital gown) on the commode. The HCA turns to me (KF) – ‘I get to the patients’ preferences!’ They are very kind and gentle with her and as they are helping her to sit on the commode, a medical team (two juniors, who appear to be Foundation Doctors) arrive and stand directly in front of the patient as she is sitting on the commode about to be wheeled out of the bay. They ignore the bay team and loom over the patient and ask her, ‘Has the stroke doctor been to see you yesterday?’ She looks up at them but does not reply. ‘There is an infarct showing in your scan so I think they will be moving you to the stroke unit’. The patient is still looking up at them but does not respond to this news and they leave. The bay team wheel her away and the nurse explains what the medical team mean in simple language and reassures her – she has clearly no idea what they were saying and looks blankly at them. [Site D day 1]

This illustrates how continence care is both mundane everyday work and has its own organizational features and routines within the ward. Patients can quickly lose control over how people see them. Note how the hierarchical structure of the ward assists this loss of control. The higher status of the medical team enables them to impose their own priorities and agency on this encounter and ignore the agency and needs of others. This demonstrates the complex, multiple nature of patients’ subordinate role in the ward's dominance hierarchies. People living with dementia are typically unable to prioritize, retain individuality or control over their own actions within this setting of an acute ward.

#### 4.1.3 An excluding barrier to social belonging

Although continence care can be invisible (as above), it can also disrupt a patient's place in the ward. Once a person is enclosed at the bedside by the curtain (to use a bedpan, disposable urinals (a ‘bottle’) or a commode), although this is a thin barrier (it is neither soundproof nor a barrier to smells), it is symbolically very powerful. Care behind the curtain is usually seen as care that must not to be interrupted and while behind the curtain on their own, a patient is also rendered invisible. However, this means that patients can quickly become excluded from important social rituals within the ward. For example, the tea trolley arriving is very powerful and important moment in the life of the ward and for socialization, stimulation and an important element of social life for patients, who may miss out:

The tea trolley comes round to the bay and a young woman is doing the tea round. She is smiley and very friendly and looks all the patients in the eye as she talks to them and makes an effort to communicate with each in turn asking their preferences.

‘Hello would you like tea or coffee?’

Bed 1 – ‘Tea, no sugar.’

‘Cup of tea or coffee?’

Bed 2 – ‘Coffee.’

‘Sugar?’

Bed 2 – ‘Sugar please.’

The patient in Bed 3 is behind the curtain [sitting on the commode] so she leaves it; she does not call out to the patient or ask the team who are with her, but moves on to the next patient,

Bed 4 – ‘Hello tea or coffee’? [Site D day 11]

It may be that the tenuous privacy afforded by the flimsy curtain barrier means that compensating measures are taken to protect privacy, measures which further exclude the person living with dementia from the social life of the ward.

### 4.2 The essential nature of continence work overrides agency

During continence work (and other body work) for people living with dementia, it was typical for ward staff (across all sites) to start with rationalization and talking to the person as having agency, but then to override it to achieve their aims and provide essential continence care. In the first extract below (Site A day 4) the HCA goes from implying choice, ‘can I?’, ‘do you want to?’ and ‘can you?’ to quickly enforcing this fundamental task that must be completed with ‘I need you to’ and ‘we need to’ to complete the task and ensure the patient is ‘decent’. Similarly, the second extract (Site D day 5) starts with ‘do you mind if I?’, which transforms into ‘I must’.

#### 4.2.1 Maintaining acceptable social presence

During continence work, there is also an emphasis on the importance of the presentation of the person to the outside world. In this example, the ward requires a patient's body to be covered and ‘decent’, and not exposed, to preserve his dignity, but also the dignity of others in the bay, staff and patents. When they draw back the curtain surrounding his bed and hiding their work from view, he is sitting in bed dressed in fresh pyjamas and covered in fresh sheets:

The HCA is busy getting the bed‐changing trolley together. She wheels the trolley and the red plastic bag fitted onto a metal frame for the soiled laundry into the bay. All the men in here seem very frail and it is silent in the bay as she sets up the morning routine. The Nurse is with Bed 5 (an 86 year old man with a diagnosis of dementia who has recently been admitted with a hematoma following a fall at home), ‘Can I just give you these tablets?’ as she does this she draws the curtain round his bed ‘Do you want to go to the toilet? There's a bottle there,’ (they repeat this to him a number of times). The HCA joins her and takes the trolley and some disposable pads behind the curtain. The nurse moves on with the medication round to the next bed and calls to the HCA behind the curtain, ‘How are you doing in there? HCA replies loudly ‘he's done a bit’ (peed in the disposable bottle). As she works behind the curtain she talks to him all the time using his first name repeatedly ‘A…, A…, A…… .do you want to pull your pants up?…you're not decent yet, A… you're not decent, can you pull your pants up, you don't want to lie there like that pal, pull your pants up, I will help you. I need you to pull up your pants, you can't lie there like that, you are all exposed, roll over that way mate, that's it’. She has kept a calm, friendly and straightforward tone through this encounter – gentle and calm. From behind the curtain, she calls to nurse – ‘Can you give me a hand? We need to get these trousers up’. When they finish, they draw back the curtain and he is propped up in the bed in hospital issue pyjamas and the sheets are tucked neatly around him. The ward is very quiet. There is only the sound of the laundry trolleys and bags from the other bays in the ward being rolled down the corridor. [Site A day 4]

While carrying out this work on his body, the HCA continues to talk to and focusses on him and there is recognition of the person she is caring for. The HCA talks to him in a style ‘come on mate’ and ‘pal’ that is levelling and contributes to normalizing this intimate body work. Here, the appropriateness of language is obviously very specific to the personal, cultural, and regional context of this encounter.

#### 4.2.2 Everyday dignity work as the enactment of agency even in its possible absence

Here the HCA keeps talking to the patient and demonstrating her recognition of him and giving all possible options as she works on his body. However, this is a charade of agency and she quickly and smoothly moves from asking him if she can perform care tasks on his body ‘Do you mind if I’, to emphasizing that these tasks must be completed ‘I must’: a nod to agency but always a return to continence as essential care:

The HCA is with the patient in a side room (a 77 year old man with a diagnosis of dementia who has had multiple falls) ‘Do you want to use the commode? Shall I check your pad? Is it wet? Shall I check your pad’? She leaves the room and comes back wheeling the commode into his single room. The HCA ‘Would you like to sit on the commode? Sit on the toilet? Do you mind if I check your pad and see if it is dry?’

Patient: HEY

HCA: Feet up.

Patient: HEY

HCA: I must check it.

Patient: HEY HEY HEY

HCA: She moves the trolley away and closes the curtain and the door ‘I will check on your pad and if it is wet I will change it’.

Patient: HEY HEY HEY HEY HEY

The HCA closes the door and from the corridor I (KF) can hear the patient as she carries out this work.

Patient: HEY HEY HEY HEY…HEY HEY HEY AAAAGGH HEY and he continues to groan loudly until she opens the door

[…] Later that afternoon a HCA arrives to relieve her and she starts the handover and reports – ‘He has just been changed but he hasn't opened his bowels for 5 days. He didn't eat anything, he ate some cornflakes, I have recorded everything’. She finishes the paperwork in the bedside file and asks him – ‘Are you tired? Would you like to go to bed?’

Patient: HEY

HCA: Would you like to go to bed?

Patient: YES

The two HCAs get the walking frame and help him up – ‘How are you today’?

[Site D day 5]

This person has a diagnosis of dementia and has limited speech, but the HCA keeps up the appearance of normality, engaging in a running commentary of her planned actions. This also reflects the difficulty for her of knowing how much he understands of this encounter and her expectations.

#### 4.2.3 The surveillance of continence can undermine agency

There is ongoing surveillance and recording of continence care and continence needs by ward staff. This can result in staff questioning a person's agency and their ability to recognize their own bodily signs and respond appropriately. During a hospital admission individuals may start to question their own bodily knowledge and their ability to recognize their bodily needs. Staff present the patient with their bedside records that are seen as external objective knowledge of their body. This can lead them to question the person about whether their own bodily feelings are valid and correct:

The patient (82 year old woman with a diagnosis of dementia who had a fractured hip) is sitting at the dining table after lunch. She looks very smart wearing a cream blouse, a black pencil skirt and leopard print slippers. She says ‘I think I want to go to the toilet, it takes me a long time to do’. The nurse responds – ‘I will do your dressing (she has thin skin on her arm that has ruptured) after, I will go and see if we can give you something (for her constipation)’. She heads off to the medicines trolley and comes back with a small pill pot – ‘this will help with your bowels’. The patient takes the tablet from her and picks up a dirty spoon from the lunch tray to eat it, but the nurse is horrified and quickly stops her and brings her a fresh dessert and spoon to eat with the tablet.

They continue the discussion about whether she wants to go to the toilet and the nurse checks in her bedside notes and reports to the patient that she has already had a bowel movement today. Now she is not sure if she does need to go or if the pain has moved to her back. They discuss this for some time and decide to ‘give it a go’. The nurse helps her stand and guides her to the commode at her bedside behind the screen. [Site D day 3]

Continence care in this context is reliant on both the routines of the ward and the administrative recordings that serve as an official version of bodily events. The patient's self‐knowledge of their bodily needs can quickly be questioned and overridden by the timing of ward routines and by what has been recorded.

### 4.3 Dignity under threat

The coexistence of a diagnosis of dementia and needing continence care creates a double jeopardy not only for the dignity and moral status of the patient, but also for those involved in their most intimate care, who must complete the pressing routines and timetables of medication rounds, personal care and continence care etc. throughout a shift. This sets the conditions within which the loss of personhood and dehumanization of people living with dementia can flourish.

#### 4.3.1 Spreading threats to dignity

In the encounter below, the person (who has a diagnosis of dementia, had a fractured hip and is now medically well and waiting for a care home placement) has been lying in her bed for all the shift. It takes a while for the team to identify that she has spread faeces onto the wall. When the team respond, she is resistant, but they work hard to reclaim dignity. Everyone's dignity is precarious in the context of continence care, their indignity of cleaning this, but also hers because she is doing something that she would not have done prior to her cognitive impairment. This demonstrates the double impact of dementia and incontinence on identity – the staff continually remind her during the encounter who she is, that this behaviour is a symptom of her condition ‘you are sick’ and they continue to focus on cleaning her hands. As they work they also continually remind her of who they are: ‘we are not servants’, which demonstrated the precarious nature of staff identity during continence care:

The patient has a corner bed in the room, she is lying on the bed surrounded by crumpled sheets – there is shit smeared on the wall her bed is against. It takes the team a while to spot this and then go over and close the curtain around her. The nurse and HCA have put plastic aprons and gloves on. They are behind the curtains with her and I (KF) can hear the sounds of them starting to clean the wall around her and as they work the HCA explains to the patient what she is doing. The patient shouts at them – ‘GO!’ The team keep talking to her ‐ ‘Don't hit us, we are helping you, we are not hurting you’. The team giggle, but it sounds like they are quite stressed and exasperated and there is the sound of lots of things being moved around behind the screen ‐ ‘You are sick’… ‘Give us your hands so we can clean them’. The HCA says in a loud voice (possibly for my benefit) ‘She's hitting me again, hitting me’… ‘No’. In response, the patient is clearly not happy ‘LEAVE ME ALONE’. The team continue with the task in hand and keep trying to clean her hands ‘give me this hand’. The patient sounds angry and retorts ‘(son's first name) is coming, she is taking everything’. The nurse comes out from behind the curtain carrying a huge pile of disposable wipes and continence pads covered in shit. The HCA reminds her ‘(first name) you are in hospital not at home’

Patient: ‘Get your hands off me, (son) is coming…he knows… . stop it.’

HCA: ‘(first name)] don't pinch us, we aren't doing it to you are we’!

Patient: ‘I will get (son) when he comes.’

Nurse: ‘We are not servants, we are your nurses.’

They are all behind the curtain and a horrible smell of shit is now filling the large room. They wheel out a large trolley with a large plastic bag full of soiled linen and pull the curtain back. She is now reclining lying fairly flat in bed wearing a clean hospital gown and a large number of diamante bracelets on her wrists, her hands are resting behind her head and she looks relaxed.

[Site D day 5]

#### 4.3.2 Dementia and the loss of options for normalized toileting

There was often the assumption that people living with dementia do not require the privacy of the bathroom and that a more limited range of options is acceptable. Here the nurse assumes that using a commode at the bedside is the appropriate response for this person. She has not checked his medical records (handover or bedside notes), nor asked the person, nor the family to establish his walking ability or his preferences. However, the family hold on to his dignity through working to maintain his normalized status of going to the bathroom (even though he has a catheter) as long as possible:

The patient (who is a 74 year old man who has chronic obstructive pulmonary disease) has his family with him, a middle aged man and woman. They tell the nurse that he has asked to go to the toilet and she replies that she will get the commode for him. They say that he has asked to go to the toilet with help. The nurse looks surprised and they explain that he can make it to the bathroom with help. The nurse and the student nurse bring a ‘steady’ (a steady frame or standing aid) for him ‘we are coming to help you up’ and spend time making sure he is able to stand and work out where on the frame to clip his catheter bag. He stands on this and they wheel him to the bathroom. [Site B day 15]

The presence of his family means that this person is able to go to the bathroom. However, this is a compromise, the ward team do not help him to walk to the bathroom, but wheel him there, which emphasizes his lack of independence.

#### 4.3.3 Recognition of continence needs demonstrates its mundane importance

The routinized but invisible nature of continence care means that staff generally do not receive recognition for this work nor realize how important this is for patients. Here the HCA is startled by the patient thanking her:

The nurse and HCA are busy behind the curtains with the patient (a 99 year old man with a fractured hip; he is also classified as having deafness and is blind) and are helping him to sit on the bed pan. They come out from behind the curtains and move on to the other patients. The patient can be heard making straining noises behind the curtain. When the HCA returns she goes straight away to see him behind the curtains. She leaves to gather fresh sheets and water and gets the things ready to wash and change him. When she has finished she pulls the curtain back to reveal him sitting up in bed in fresh hospital pyjamas and with a fresh sheet over him and the trolley over the bed (just the way he likes it). The HCA goes back to check on him and gives him some tissues. He calls her over again and asks her if she was the one who helped him ‘Did you help me’? She is surprised and says yes. Patient: ‘Thank you very much, you are a genius!’ HCA: ‘Thank you!’ (she looks very surprised). She looks at me and we laugh together about this later. [Site A day 5]

Continence care has little recognition in the ward, however, moments like this demonstrate how important it is for the person.

## 5 DISCUSSION

Continence is a key moment of care that can tell us about the wider care of people living with dementia within acute hospital wards. The challenge of continence care for people living with dementia can be seen as the ‘canary in the coal mine’ for the unravelling of dignity. However, by this very fact, continence care is a locus for sensitive and creative care work which can protect, maintain, and rehabilitate dignity.

In the space of this paper, we have not been able to do more than indicate how our findings suggest that personal dignity and the social construction of moral personhood are both threatened and maintained in such a setting. However, there are some telling observations which we propose show how empirical ethnographic data can lend weight to, and add detail to, theoretical accounts of moral personhood and dignity.

The fragility of mental and bodily agency that comes with a diagnosis of dementia and (often assumed) poor continence control, means that steps must be taken to preserve a patient's place in the moral community as a person meriting dignity. Within the routinized and hierarchical setting of a hospital ward, any such steps must operate within the constraints of the system, a system which perhaps acts as a vivid microcosm of the wider social world. Losses to dignity are imposed by routines and dominance hierarchies which disrupt an individual's control over the visibility of their elimination needs (that others can freely assume to have), and disrupt the presence of the individual in the social life of the ward. Our analysis identified that people living with dementia were often acutely aware of their loss of control. This was not only their loss of bodily control, but was often also the loss of control that is eroded by the ward routines and timetables that they must submit to.

The threats to selfhood and agency are seen even in the ways that the necessity of continence care within wards may be used to question or override a person's own basic self‐knowledge of the body's promptings of bladder or bowel. Such questioning of self‐knowledge may be necessitated by the routines and pace of hospital work. This indignity spreads to all who are in contact with the patient experiencing difficulties with continence; to staff doing ‘body work’, who are already of low status, and to relatives. Hence, those rescuing the moral dignity of the person are those whose social status is further threatened by their contact with bodily wastes.

Attempts to counter these threats to dignity included attempts to impose an appearance of controlled order in the presentation of the person to others. Yet attempts to ignore the social incongruity of toileting through a ‘pretence of invisibility’ may act to deepen loss of dignity (for example where important medical results are communicated to a patient being wheeled to the toilet) and to increase the sense of isolation (being behind a thin screen may mean that compensating measures are taken, but which further exclude the person with dementia from the social life of the ward). We propose that such socially chaotic disruption to expectations of the visible and the invisible lies at the heart of understanding the indignity to which these people are exposed. The status of different personnel, and the imposition of ward routines, create a complex mix within which threats to dignity must be understood.

We suggest, as have many others,^52^ that further work needs to be undertaken to examine how these factors interact in threatening and reconstructing moral personhood and dignity. The body of work to date examining dignity amongst older people living at home or in long‐term community settings has produced findings that resonate with many of ours.^53^ For example, the importance of appearance in maintaining dignity,^54^ the importance of inclusion in social occasions such as mealtimes,^55^ and the way in which the capacity to provide dignified care impacts on the dignity of staff.^56^ Our approach emphasizes that dignity is something that is to be found between people and emerges in the concrete interactions within a particular social setting. The erosion of dignity and threats to personhood cannot be understood simply as a result of primary losses of mental and bodily control of the patients. Nor can they be understood simply in terms of stigmatized responses or the disgust of others. It needs to be recognized that stigma and indignity are socially contagious. We need to understand the complex ways in which the presence in the social world of that which should not be present is negotiated, and how such negotiation can ameliorate or worsen the situation. This can all only fully be understood by examining the complex systems of dominance, hierarchy, and social and institutional routines.

## CONFLICT OF INTEREST

The author declares no conflict of interest.

## Footnotes

1
The House of Lords (2007). *The human rights of older people in healthcare,* Eighteenth Report of Session 2006–07 Report and Formal Minutes Ordered by the House of Commons. London: House of Commons; Francis, R. (2013). *Report of the Mid Staffordshire NHS Foundation Trust Public Inquiry: Executive Summary* London; Boaden, A. (2016). *Fix dementia care: hospitals*. Alzheimer's Society: London.

2
Care Quality Commission (2011). *Dignity and nutrition inspection programme: National overview*. London: Care Quality Commission.

3
Phelan, E. A., et al. (2012). Association of incident dementia with hospitalizations. *JAMA*, *307*, 165–172. 10.1001/jama.2011.1964PMC331292122235087

4
Sampson, E. L., et al. (2009). Dementia in the acute hospital: Prospective cohort study of prevalence and mortality. *Br J Psychiatry, 195*, 61–66. 10.1192/bjp.bp.108.05533519567898

5
Ibid.

6
Pinkert, C., & Holle, B. (2012). People with dementia in acute hospitals: Literature review of prevalence and reasons for hospital admission. *Z Gerontol Geriatr, 45*, 728–734. 10.1007/s00391-012-0319-122538786

7
Holmes, J., & House, A. (2000). Psychiatric illness predicts poor outcome after surgery for hip fracture: A prospective cohort study. *Psychol Med, 30*, 921–929. 10.1017/s003329179900254811037100

8
Boaden, *op. cit*. note 1.

9
Morris, J. (2007) Behind closed doors. *Work Older People, 11*(2), 35–38.

10
The House of Lords, *op. cit* note 1; Patients Association (2009). *Patients… not numbers, people… not statistics. Patients Association*; Department of Health (2006). *A new ambition for old age: next steps in implementing the national service framework for older people. A report from Professor Ian Philp, National Director for Older People* London: Department of Health; Nottingham City and Nottinghamshire County Joint Health Scrutiny Committee (2010). *Review of dementia care in hospitals: Report of the Nottingham City and Nottinghamshire County Joint Health Scrutiny Committee*. Nottingham City and Nottingham County: Nottingham.

11
Francis, *op. cit*. note 1.

12
The House of Lords, *op. cit*. note 1.

13
Patients Association, *op. cit* note 10; The House of Lords, *op. cit* note 1; Department of Health, *op. cit*. note 10.

14
Bridges, J., et al. (2013). Capacity for care: meta‐ethnography of acute care nurses' experiences of the nurse‐patient relationship. *J Adv Nurs, 69*, 760–772. 10.1111/jan.12050PMC361746823163719

15
Bridges, J., Flatley, M., & Meyer, J. (2010). Older people's and relatives’ experiences in acute care settings: systematic review and synthesis of qualitative studies. *Int J Nurs Stud*, 47, 89–107. 10.1016/j.ijnurstu.2009.09.00919854441

16
Bridges, J., & Wilkinson, C. (2011). Achieving dignity for older people with dementia in hospital. *Nurs Stand*, *25*(29), 42–47. 10.7748/ns2011.03.25.29.42.c840221500689

17
Lloyd, J. V., et al. (2011). Ingroup identity as an obstacle to effective multiprofessional and interprofessional teamwork: findings from an ethnographic study of healthcare assistants in dementia care. *J Interprof Care, 25*, 345–351; Daykin, N., & Clarke, B. (2000). ‘They'll still get the bodily care’. Discourses of care and relationships between nurses and health care assistants in the NHS. *Sociol Health Illn, 22*, 349–363. 10.3109/13561820.2011.56738121635181

18
Daykin & Clarke, *op. cit*. note 17.

19
Wolkowitz, C. (2006). *Bodies at work*. London: Sage.

20
Twigg, J. (2000). Carework as a form of bodywork. *Ageing Soc*, *20*, 389–411.

21
Simpson, R., Slutskaya, N., & Hughes, J. (2012). Gendering and embodying dirty work: Men managing taint in the context of nursing care. In R. Simpson et al. (Eds) *Dirty work: Concepts and identities*. Basingstoke: Palgrave MacMillan UK.

22
Twigg, J. *op. cit* note 20; Bolton, S. C. (2005). Women's work, dirty work: The gynaecology nurse as ‘other’. *Gend Work Organ*, *12*, 169–186

23
Jervis, L. L. (2001). The pollution of incontinence and the dirty work of caregiving in a US nursing home. *Med Anthropol Q*, *15*, 84–99. 10.1525/maq.2001.15.1.8411288620

24
Lawler, J. (1991). *Behind the screens: Nursing, somology, and the problem of the body*. Melbourne, Edinburgh: Churchill Livingstone.

25
Lee‐Trewick, G. (1994). Bedroom abuse: the hidden work in a nursing home. In J. Johnson & C. DeSouza (Eds.) *Understanding health and social care an introductory reader* (2nd ed., pp. 107—111) London: Sage:

26
Heath, H. (2009). The nurse's role in helping older people to use the toilet. *Nurs Stand*, *24*(2), 43–47. 10.7748/ns2009.09.24.2.43.c726619813407

27
Downie, R. S., & Telfer, E. (1969). *Respect for persons*. London: Allen and Unwin.

28
Davidson, D. (1980). *Essays on actions and events* Oxford: Clarendon Press; Hornsby, J. (1980). *Actions*. London: Routledge & Kegan Paul.

29
Wasserman, D., et al. (2017). Cognitive disability and moral status. In E. N. Zalta (Ed.) *The Stanford encyclopedia of philosophy*. Stanford, CA: Stanford University.

30
Iser, M. (2013). Recognition. In E. N. Zalta (Ed.) *The Stanford encyclopedia of philosophy* Stanford, CA: Stanford University; Sandel, M. J. (1998). *Liberalism and the limits of justice* Cambridge: Cambridge University Press; Taylor, C. (1991). *The ethics of authenticity* Cambridge, MA: Harvard University Press; Aristotle (1999). *Nicomachean Ethics*. (2nd ed. T. Irwin, trans.) Indianapolis, IN: Hackett Publishing Co. Inc.

31
Honneth, A. (1996). *The struggle for recognition: the moral grammar of social conflicts* Boston, MA: MIT Press; Butler, J. (2015). *Notes toward a performative theory of assembly*. London: Harvard University Press.

32
Tranvåg, O., Petersen, K. A., & Nåden, D. (2016). Dignity‐preserving dementia care: a metasynthesis. *Nurs Ethics*, *20*, 861–880. 10.1177/096973301348511023715554

33
Nordenfelt, L. (2004). The varieties of dignity. *Health Care Anal*, *12*, 69–81. 10.1023/B:HCAN.0000041183.78435.4b15487812

34
Gallagher, A., et al. (2008). Dignity in the care of older people – a review of the theoretical and empirical literature *BMC Nurs, 7*, 11. 10.1186/1472-6955-7-11PMC248398118620561

35
Zimbardo, P. (2007). *The Lucifer effect: Understanding how good people turn evil*: London: Rider; Milgram, S. (1974). *Obedience to authority: an experimental view*. New York: Harper and Row.

36
Zimbardo, *op. cit*, note 35; Greenberg, K. J., & Dratel, J. L. (2005). *The torture papers: the road to Abu Ghraib*. New York: Cambridge University Press.

37
Kane, J., & de Vries, K. (2017). Dignity in long‐term care: an application of Nordenfelt's work. *Nurs Ethics*, *24*, 744–751. 10.1177/096973301562448726811400

38
Franklin, L. ‐L., Ternestedt, B. ‐M., & Nordenfelt, L. (2006). Views on dignity of elderly nursing home residents. *Nurs Ethics, 13*, 130–146. 10.1191/0969733006ne851oa16526148

39
Naue, U., & Kroll, T. (2009). The demented other: identity and difference in dementia, *Nurs Philos*, *10*, 26–33. 10.1111/j.1466-769X.2008.00379.x19154294

40
Martin, R., & Barresi, J. (2003). *Personal identity and what matters in survival: an historical overview*. Oxford: Blackwell.

41
Edvardsson, D., Fetherstonhaugh, D., & Nay, R. (2010). Promoting a continuation of self and normality: person‐centred care as described by people with dementia, their family members and aged care staff. *J Clin Nurs*, *19*, 2611–2618. 10.1111/j.1365-2702.2009.03143.x20586833

42
McCormack, B. (2004). Person‐centredness in gerontological nursing: an overview of the literature. *J Clin Nurs*, *13*(s1), 31–38; Heggestad, A. K. T., Nortvedt, P., & Slettebø, Å. (2015). Dignity and care for people with dementia living in nursing homes. *Dementia*, *14*, 825–841. 10.1177/147130121351284024381212

43
Tranvåg et al. *op. cit*. note 32.

44
Heggestad, A. K. T., Nortved, P., & Slettebø, Å. (2013) ‘Like a prison without bars’ dementia and experiences of dignity. *Nurs Ethics, 20*, 881–892. 10.1177/096973301348448423702888

45
Nåden, D., et al. (2013). Aspects of indignity in nursing home residences as experienced by family caregivers. *Nurs Ethics, 20*, 748–761. 10.1177/096973301247525323462504

46
Brooker, D. (2007). *Person‐centred dementia care: making services better* London: Jessica Kingsley Publications; Leadbeater. C. (2015). Where does identity go once memory falters in dementia? *Aeon*, March 2015, http://aeon.co/magazine/psychology/where-does-identity-go-once-memory-falters-in-dementia/

47
Atkinson, P., & Housley, W. (2003). *Interactionism*. London: Sage.

48
Hammersley, M. (1987). *What's wrong with ethnography? Methodological explorations*. London: Routledge.

49
Atkinson, P., Delamont S., & Housley, W. (2008). *Contours of culture: complex ethnography and the ethnography of complexity*. Altamira: Rowman.

50
Marshall, M. N. (1996). Sampling for qualitative research. *Fam Pract*, *13*, 522–526. 10.1093/fampra/13.6.5229023528

51
Glaser, B., & Strauss, A. (1967). *The discovery of grounded theory*. London: Weidenfeld and Nicholson.

52
Gallagher, A. (2011). What do we know about dignity in care? *Nurs Ethics, 18*(4), 471–473. 10.1177/096973301141384521788285

53
Gallagher, A., et al. (2008). Dignity in the care of older people – a review of the theoretical and empirical literature. *BMC Nurs, 7*, 11; Nuffield Council On Bioethics (2009). *Dementia: ethical issues*. London, England: Nuffield Council on Bioethics. 10.1186/1472-6955-7-11PMC248398118620561

54
Morgan, K. (2011). Dignity in dementia: a personal view. *Dementia, 10*, 281–282.

55
Nåden et al., *op. cit*. note 45.

56
Dwyer, L. L., Andershed, B., Nordenfelt, L., & Ternestedt, B. M. (2009) Dignity as experienced by nursing home staff. *Int J Older People Nurs, 4*, 185–193. 10.1111/j.1748-3743.2008.00153.x20925775

